# RAD-Seq and Ecological Niche Reveal Genetic Diversity, Phylogeny, and Geographic Distribution of *Kadsura interior* and Its Closely Related Species

**DOI:** 10.3389/fpls.2022.857016

**Published:** 2022-04-26

**Authors:** Yuqing Dong, Xueping Wei, Tingyan Qiang, Jiushi Liu, Peng Che, Yaodong Qi, Bengang Zhang, Haitao Liu

**Affiliations:** ^1^Key Laboratory of Bioactive Substances and Resources Utilization of Chinese Herbal Medicine, Ministry of Education, Institute of Medicinal Plant Development, Chinese Academy of Medical Sciences, Peking Union Medical College, Beijing, China; ^2^Engineering Research Center of Tradition Chinese Medicine Resource, Ministry of Education, Institute of Medicinal Plant Development, Chinese Academy of Medical Sciences, Peking Union Medical College, Beijing, China

**Keywords:** divergence, ecological niche, genetic relationship, *Kadsura interior*, population structure, RAD-seq, SNP

## Abstract

Most plants of *Kadsura* have economic value and medicinal application. Among them, *K. interior* and its closely related species have been demonstrated to have definite efficacy. However, the taxonomy and phylogenetic relationship of *Kadsura* in terms of morphology and commonly used gene regions remain controversial, which adversely affects its rational application. In this study, a total of 107 individuals of *K. interior*, *K. heteroclita*, *K. longipedunculata*, *K. oblongifolia*, and *K. coccinea* were studied from the perspectives of genetic diversity, phylogeny, and ecology *via* single nucleotide polymorphisms (SNPs) developed through restriction site-associated DNA sequencing (RAD-seq). Based on these SNPs, the genetic diversity, phylogenetic reconstruction, and population genetic structure were analyzed. Subsequently, divergence time estimation and differentiation scenario simulation were performed. Meanwhile, according to the species distribution records and bioclimatic variables, the Last Glacial Maximum and current potential distributions of five species were constructed, and the main ecological factors affecting the distribution of different species were extracted. The *F*_ST_ calculated showed that there was a moderate degree of differentiation among *K. heteroclita*, *K. longipedunculata*, and *K. oblongifolia*, and there was a high degree of genetic differentiation between *K. interior* and the above species. The phylogenetic tree indicated that each of the species was monophyletic. The results of population genetic structure and divergence scenario simulation and D-statistics showed that there were admixture and gene flow among *K. heteroclita*, *K. longipedunculata*, and *K. oblongifolia*. The results of ecological niche modeling indicated that the distribution areas and the bioclimatic variables affecting the distribution of *K. interior* and its related species were different. This study explored the differences in the genetic divergence and geographical distribution patterns of *K. interior* and its related species, clarifying the uniqueness of *K. interior* compared to its relatives and providing a reference for their rational application in the future.

## Introduction

Phylogenies are important for addressing various biological questions such as relationships among species and histories of populations (Kapli et al., 2020). Understanding the relationships among species is an important goal and the foundation of identification as well as further application (Yang and Rannala, 2012). At present, we are focusing on *Kadsura*, which is faced with problems of phylogenetic confusion and species delimitation. *Kadsura*, belonging to Schisandraceae, consists of about 16 species worldwide, and around eight of its species are distributed from the southeast to the southwest of China (Xia et al., 2008). Most of the plants in this genus are used for medicinal purposes and have good activities of anti-inflammatory, antitumor, and antioxidant biological activities (Liu et al., 2018; Sritalahareuthai et al., 2020). Previous studies found that *Kadsura interior*, *K. heteroclita*, *K. longipedunculata*, and *K. oblongifolia* are very closely related. Until now, their phylogenetic relationship has remained unclear. Zhang et al. (2015) reconstructed the phylogenetic tree of Schisandraceae based on four commonly used gene regions (ITS, *psb*A-*trn*H, *mat*K, and *rbc*L) and found that the samples of *K. heteroclita* and *K. longipedunculata* intersected and could not be discriminated. Guo et al. (2017) used the same gene regions to identify *K. interior* from its closely related species and found that the samples of *K. interior*, *K. heteroclite*, and *K. longipedunculata* were mixed and clustered into one clade. This phenomenon may also be related to hybridization or introgression. Hybridization and introgression among related species have transformed the understanding of phylogenetic trees from strictly bifurcating trees to reticulate trees (Mallet et al., 2016). Unraveling the hybridization process can help us understand the origin of species and better resolve phylogenetic relationship. The evolution analyses at the population level can provide strong evidence for understanding the phylogenetic relationships and reveal potential hybridization events of closely related species (Zhou et al., 2020). What exactly is the phylogenetic relationship of these species? Did they experience hybridization events during speciation? Further studies are needed to be discussed.

In China, the stems of *K. interior*, known as “Dian Ji Xue Teng,” are officially recorded in the current Chinese Pharmacopoeia and used to tonify and invigorate blood in traditional Chinese medicine (TCM) (Yin et al., 2017; Chinese pharmacopoeia commission, 2020). The stems of *K. heteroclita*, *K. longipedunculata*, *K. oblongifolia*, and *K. coccinea* are also documented as TCM and have some different efficacies in the folk (Guangxi Zhuang Autonomous Region Health Department, 1992; Guangdong Food and Drug Administration, 2004; Fujian Food and Drug Administration, 2006). And in the treatment of the tonify and invigorate blood, the effect of “Dian Ji Xue Teng” is significantly better (Xu et al., 2022). Due to the similar morphological characteristics, Saunders and Lin treated *K. interior* as the synonym *K. heteroclita*, and the Flora of China adopted this treatment, which might led to misuse in folk (Saunders, 1998; Lin, 2002; Xia et al., 2008). The misuse caused by unclear species relationships and delimitation may affect the therapeutic efficacy and even cause safety issues (Wu et al., 2007; Xu et al., 2022). Therefore, clarifying the species delimitation and interspecies relationship is crucial to their use.

As the whole genome of *Kadsura* has not yet been sequenced, and the genome of the is around 7.36 G, which makes it difficult to conduct research through the whole genome (Xu et al., 2021). RAD-seq, a high-throughput sequencing technology, enables the development of large-scale SNPs without relying on the reference genomes to provide greater phylogenetic resolution than conventional gene regions (Miller et al., 2007). It is advantageous especially when the sample size is large, thus having been widely used in population genetic research, especially in the phylogenetic studies of populations or closely related species (Paetzold et al., 2019; Boukteb et al., 2021).

The purpose of this study is to investigate genetic diversity, interspecific phylogenetic relationship, and population genetic structure of *K. interior* and its related species by RAD-seq. At the same time, the divergence time of *K. interior* and its relative was estimated through SNPs, combined with geohistorical events, to infer the cause of species divergence. In addition, ecological niche modeling was used to explore the potential distribution differences and niche differentiation of these species.

## Materials and Methods

### Sample Collection and Processing

**FIGURE 1:**
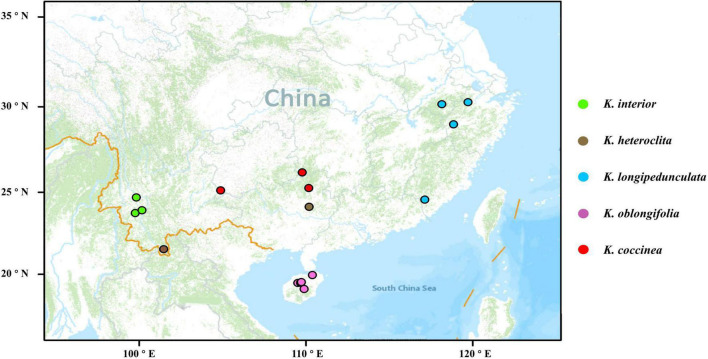
Sample distribution of the five species of *Kadsura.* The base map was generated by ArcGIS v10.7.

In previous studies, *K. interior*, *K. heteroclita*, *K. longipedunculata*, and *K. oblongifolia* clustered as a clade, and the other species of the *Kadsura* were more distantly to these species. We collected samples of *K. interior*, *K. heteroclita, K. longipedunculata, K. oblongifolia*, and one species *K. coccinea* as outgroup in the south of the Yangtze River, with a total of 107 individuals from 17 populations, covering the main distribution areas of these five species (Figure 1). For each population, the individuals we sampled were more than 50 m apart as far as possible, and almost all populations contained more than five individuals. Fresh leaves were cleaned and dried in silica gel in the field, or stored in dry ice and placed in the refrigerator at −80°C in time.

### DNA Extraction and Sequencing of RAD Libraries

Total genomic DNA was extracted using a Plant Genomic DNA kit (TianGen, Beijing, China) following the manufacturer’s instructions. The detection of DNA degradation and contamination was done with 1% agarose gels and the DNA concentration was measured by Qubit^®^ DNA Assay Kit in Qubit^®^ 3.0 Fluorometer (Invitrogen, United States). The qualified DNA was digested by *Eco*RI, and P1 adapters were added at the ends of the digestion fragments. These fragments are subsequently pooled, randomly sheared, and size-selected. Then, the P2 adapter with a special “Y-type” structure was attached to the DNA fragments, ensuring that PCR only amplifies the sequences with two adapters at the same time. The quality of the libraries was checked by Qubit kit, Agilent2100, and Q-PCR. The qualified libraries were sequenced by the Illumina Hiseq platform to generate 150 bp paired-end reads (Miller et al., 2007; Baird et al., 2008). At present, there is no complete genome of the *Kadsura* species, so the *K. interior* genome size estimated by flow cytometry (FCM) is used as a reference for sequencing the depth (Xu et al., 2021).

### Single Nucleotide Polymorphism Calling and Genetic Diversity

STACKS v2.55 software pipeline (Rochette et al., 2019) was used to call SNPs. Initially, all samples were checked for the barcode and the RAD cut site integrity, and low-quality reads were filtered out (Phred score < 10). Then, the USTACKS program was used to cluster one individual of *K. interior*, and the consensus sequence of RAD loci after clustering was extracted as the reference sequence. BWA was used to map reads of the remaining individuals to the reference sequence (Li and Durbin, 2009). The GSTACKS program was used to create loci by incorporating paired-end reads that have been aligned to the reference sequence and sorted. Meanwhile, the “–rm-pcr-duplicates” option was selected to remove the PCR duplicates. The POPULATIONS program was used to filter SNPs and generate files needed for downstream analysis. The filter parameters included the following: minimum percentage of individuals in a population required to process a locus for that population (-r), the minimum number of populations a locus must be present in to process a locus (-p), the minimum minor allele frequency required to process a nucleotide site at a locus (–min-maf), and the maximum observed heterozygosity required to process a nucleotide site at a locus (–max-obs-het). We filtered the original SNPs and produced two datasets based on the species level (*r* = 0.5, *p* = 5, min-maf = 0.05, max-obs-het = 0.7) and population level (*r* = 0.5, *p* = 17, min-maf = 0.05, max-obs-het = 0.7) (Rochette and Catchen, 2017). Based on the two datasets, the observed heterozygosity (*H*o), expected heterozygosity (*H*e), inbreeding coefficient (*F*_IS_), and pairwise fixation index (*F*_ST_) were calculated by the fstats module in the POPULATIONS program. For nucleotide diversity (π), we used BCFTOOLS to generate the VCF file for all sites, which included variant sites and invariant sites. Then, PIXY was used to calculate π at the species level and population level, respectively (Korunes and Samuk, 2021).

### Phylogeny and Population Structure Based on SNP

We performed PCA analysis using the smartpca module in EIGENSOFT based on the SNP dataset at the species level. The first 10 principal components were calculated and the first 3 principal components were visualized with the ggplot2 package in R. Then maximum-likelihood phylogenetic trees were constructed using IQTREE v2.1.4 (Minh et al., 2020). We used the built-in modelfinder to evaluate the best substitution model and finally selected TVMe + ASC + R3 as the best-fit model according to BIC (Bayesian Information Criterion) and used the ascertainment bias correction (+ ASC) model to correct the likelihood conditioned on the variable sites (Lewis, 2001; Kalyaanamoorthy et al., 2017). The bootstrap values were obtained by 1,000 bootstrap replicates. Ultimately, we identified the population genetic structure using STRUCTURE v 2.3.4 (Pritchard et al., 2000). This analysis required SNPs not to be physically linked, so we filtered the SNP dataset at the species level using the “–write-single-snp” option in the POPULATIONS program to generate independent SNPs (Ren et al., 2017). The model selected for the structure analysis was the admixture model, the length of the burn-in period was 1 × 10^5^, and the number of MCMC repetitions after the burn-in was 2 × 10^5^. The number of genetic clusters was set to range from *K* = 1 to *K* = 10 and ran independently 20 times. The optimal *K* was selected according to the delta-*K* calculated in STRUCTURE HARVESTER (Earl and Vonholdt, 2012). The results of the 20 independent runs were merged by CLUMPP and visualization using the R package POPHELPER (Jakobsson and Rosenberg, 2007; Francis, 2017).

### Chloroplast Genome Assembly and Phylogenetic Analysis

One sample from each population was selected for genomic DNA paired-end sequencing on the illumine platform. The raw data were filtered by Skewer v0.2.2 (Jiang et al., 2014). Chloroplast genome-like reads were extracted by performing a BLAST search with the reference sequence (NCBI Accession: NC_050348.1). Then, the selected reads were assembled into contigs using the assembly software ABySS v2.0 with a k-mer set of 127 (Jackman et al., 2017). The orientation and circularization of the contigs were performed by using Geneious (Kearse et al., 2012). Finally, BWA was used to map clean reads to draft chloroplast genome and ensured that each base was correct (Li and Durbin, 2009). The chloroplast genome sequences were aligned by MAFFT (Katoh and Standley, 2013), and phylogenetic trees were constructed using IQTREE v2.1.4 (Minh et al., 2020).

### Divergence Scenarios and Time Estimation

To determine the most likely divergence scenario and admixture events of these species, we considered 7 scenarios through approximate Bayesian calculations using DIY-ABC v2.1.0 based on the results of the PCA, phylogenetic, and STRUCTURE analysis (Cornuet et al., 2014). Like STRUCTURE analysis, the input data were the non-linked SNPs dataset. The scenario settings were divided into two situations: one was that there were no admixture events between species, and the other was that there were admixture events. For each scenario, we chose the uniform prior probability and considered all summary statistics to generate a reference table containing 1 × 10^6^ simulated datasets. The 1 × 10^4^ simulated datasets that were closest to the observed dataset were used to estimate posterior probabilities for each scenario *via* logistics.

D-statistic, also known as the ABBABABA test, can be used to test ancient admixture or wrong tree topology (Durand et al., 2011). We used the program doAbbababa2 of ANGSD to detect the gene flow between *K. interior* and its closely related species (Soraggi et al., 2018). We selected all bam files of the four species *K. interior*, *K. heteroclita*, *K. longipedunculata*, and *K. oblongifolia* and selected a sample of *K. coccinea* as the outgroup.

The divergence times within *Kadsura* were inferred using BEAST v2.6.4 (Bouckaert et al., 2019). The tree was calibrated at the most recent common ancestor (MRCA) of *K. coccinea* and the other species (25.2 Ma, 95% HPD: 12.2–41.9 Ma) (Fan et al., 2011). We used 5,998 non-linked SNPs and prepared the XML input file for SNAPP with the Ruby script *snapp_prep.rb* (Stange et al., 2018). We wrote the single age calibration point to a constraint file and specified a lognormal distribution with an offset of 0, a mean of 25.2, and a standard deviation of 0.35 (in real space). The Markov chain Monte Carlo (MCMC) chain length was 1 × 10^6^ and sampled every 500 generations. The effective sample size (ESS) was estimated in Tracer v.1.7.2 to be > 200 for each parameter. FigTree v1.4.4 was used for visualization.

### Ecological Niche Modeling

According to the Chinese Virtual Herbarium (CVH^1^), Global Biodiversity Information Facility [GBIF.org (13 July 2021) Occurrence Download^2^ ] and field investigations, the distribution data (longitude and latitude coordinates) of five *Kadsura* species were obtained. Then, the redundant data were removed by ENMtools v1.4. A total of nineteen bioclimatic variable layers for the present period (1960–1990) and paleoclimate (Mid-Holocene, Last Glacial Maximum) were downloaded from WorldClim v1.4^3^ with a spatial resolution of 2.5 arc min (Hijmans et al., 2005; Dupin et al., 2011). For LGM and MH, we chose three General Circulation Models (GCMs): CCSM4, MIROC-ESM, and MPI-ESM-P. And we chose an appropriate background area to run the model (85°E-135°E, 10°N-35°N). The ecological niche models were constructed with the program Maxent v3.4.1 to determine the potential geographic distribution of each species. The models were calibrated with 70% of the data and evaluated with the remaining 30%, the area under the AUC curve, and true skill statistics. The importance of each variable to the model was evaluated by the jackknife method, and the one with higher importance was reserved among the variables with a Pearson correlation > 0.8 (Hill et al., 2012). Finally, we changed the continuous distribution threshold automatically generated by the MaxEnt software to bivariate distribution (suitable area and non-suitable area), and set the threshold to Maximum Training Sensitivity plus Specificity Logistic (MTSS). At the same time, the threshold value and number 1 were divided into three equal parts, corresponding to the low, moderate, and high suitable areas, respectively. And the layer was made in DIVA-GIS v 7.5.0. For LGM and MH, the layers predicted by the three GCMs were stacked through DIVA-GIS, and then the stacked layers were averaged, and finally, the results were predicted by the three GCMs combination.

## Results

### SNP Calling and Genetic Diversity

About 976 G of clean data were generated from 107 individuals with an average of 9.12 G per sample (Table 1). The sequencing depth ranged from 8.92 × to 55.16 ×, with an average sequencing depth of 22×. We obtained 5,998 RAD loci containing 18,820 SNPs at the species level and 813 RAD loci containing 1,771 SNPs at the population level. The two SNPs datasets were used to calculate genetic diversity, respectively, and the species level SNPs dataset was used for other analyses.

**TABLE 1:** Sequencing sample information summary.

Species	Population ID	Position	Number	Longitude/°	Latitude/°	Altitude/m
*K. interior*	KI_FQ	Fengqing, Yunnan	10	99.843	24.675	2,427
	KI_GM	Gengma, Yunnan	10	99.777	23.735	2,537
	KI_LC	Lincang, Yunnan	6	100.182	23.907	2,319
*K. heteroclita*	KH_LB	Laibin, Guangxi	6	110.204	24.112	1,133
	KH_ML	Mengla, Yunnan	7	101.481	21.531	1,280
*K. longipedunculata*	KL_HS	Huangshan, Anhui	4	118.168	30.13D-0	1,582
	KL_ZZ	Zhangzhou, Fujian	5	117.144	24.548	879
	KL_QZ	Quzhou, Zhejiang	8	118.817	28.759	250
	KL_LA	Linan, Zhejiang	6	119.732	30.240	320
*K. oblongifolia*	KO_ZZ	Zhanzhou, Hainan	6	109.523	19.436	204
	KO_BL	Bailing, Qiongzhong, Hainan	6	109.907	19.063	210
	KO_HQ	Huaqiaoyidui, Qiongzhong, Hainan	6	109.694	19.456	195
	KO_DWL	Dawangling, Qiongzhong, Hainan	6	109.709	19.462	221
	KO_HK	Haikou, Hainan	6	110.399	19.935	20
*K. coccinea*	KC_HH	Huaihua, Hunan	5	109.790	26.164	421
	KC_QXN	Qianxinan, Guizhou	5	104.902	25.098	1,197
	KC_GL	Guilin, Guangxi	5	110.186	25.242	154

The average within-species nucleotide diversity (π) ranged from 0.0121 (*K. interior*) to 0.02878 (*K. coccinea*) and the average within-population π ranged from 0.0101 (KI_LC) to 0.0294 (KC_GL) (Table 2). Overall, the differences in the nucleotide diversity of these species were not significant. *K. coccinea* exhibited higher genetic diversity and *K. interior* exhibited the lowest genetic diversity. The observed heterozygosity ranged from 0.0105 (*K. interior*) to 0.0187 (*K. coccinea*) at the species level, and the expected heterozygosity ranged from 0.0760 (*K. interior*) to 0.1082 (*K. oblongifolia*). The inbreeding coefficient in each species ranged from 0.2124 (*K. coccinea*) to 0.3381 (*K. oblongifolia*). The fixation index (*F*_ST_) ranged from 0.0737 (*K. longipedunculata* vs. *K. oblongifolia*) to 0.5031 (*K. interior* vs. *K. coccinea*) at the species level (Table 3). There are three general gradients, the highest *F*_ST_ between *K. coccinea* and other species ranged from 0.4386 to 0.5031 which indicates there is a great genetic differentiation among them. Then, except for *K. coccinea*, the *F*_ST_ between *K. interior* and other species was around 0.15, indicating that there was a moderate level of genetic differentiation among them. Moreover, the *F*_ST_ among *K. heteroclita*, *K. longipedunculata*, and *K. oblongifolia* was all less than 0.1 with a very low degree of differentiation. Meanwhile, pairwise *F*_ST_ among the populations also showed a similar situation (Supplementary Table 1).

**TABLE 2:** Population statistics calculated for the RAD-seq loci.

Species	Pop ID	Π		*Ho*		*He*		*F*_IS_	
		species level	pop level	species level	pop level	species level	pop level	species level	pop level
*K. interior*	KI_FQ	0.0121	0.0113	0.0105	0.0148	0.0760	0.0421	0.2163	0.0840
	KI_GM		0.0120		0.0163		0.0498		0.0989
	KI_LC		0.0101		0.0182		0.0426		0.0680
*K. heteroclita*	KH_LB	0.0203	0.0185	0.0135	0.0230	0.0796	0.0500	0.2151	0.0751
	KH_ML		0.0190		0.0234		0.0538		0.0937
*K. longipedunculata*	KL_HS	0.0197	0.0183	0.0144	0.0275	0.0864	0.0484	0.2873	0.0581
	KL_ZZ		0.0185		0.0178		0.0497		0.0850
	KL_QZ		0.0180		0.0262		0.0589		0.1007
	KL_LA		0.0194		0.0281		0.0497		0.0689
*K. oblongifolia*	KO_ZZ	0.0179	0.0172	0.0149	0.0254	0.1082	0.0639	0.3381	0.1102
	KO_BL		0.0162		0.0266		0.0611		0.0996
	KO_HQ		0.0153		0.0250		0.0660		0.1140
	KO_DWL		0.0149		0.0232		0.0597		0.0987
	KO_HK		0.0168		0.0248		0.0645		0.1121
*K. coccinea*	KC_HH	0.02878	0.0293	0.0187	0.0346	0.0897	0.0728	0.2124	0.1042
	KC_QXN		0.0289		0.0422		0.0769		0.0984
	KC_GL		0.0294		0.0405		0.0787		0.1073

*Ho and He represent mean observed and expected heterozygosity.*

**TABLE 3:** Pairwise *F*_ST_ among *K. interior*, *K. heteroclita, K. longipedunculata, K. oblongifolia*, and *K.coccinea.*

	*K. heteroclita*	*K. longipedunculata*	*K. oblongifolia*	*K. coccinea*
*K. interior*	0.1558	0.1347	0.1446	0.5031
*K. heteroclita*		0.0820	0.0918	0.4958
*K. longipedunculata*			0.0737	0.4552
*K. oblongifolia*				0.4386

### Phylogeny and Population Structure

**FIGURE 2:**
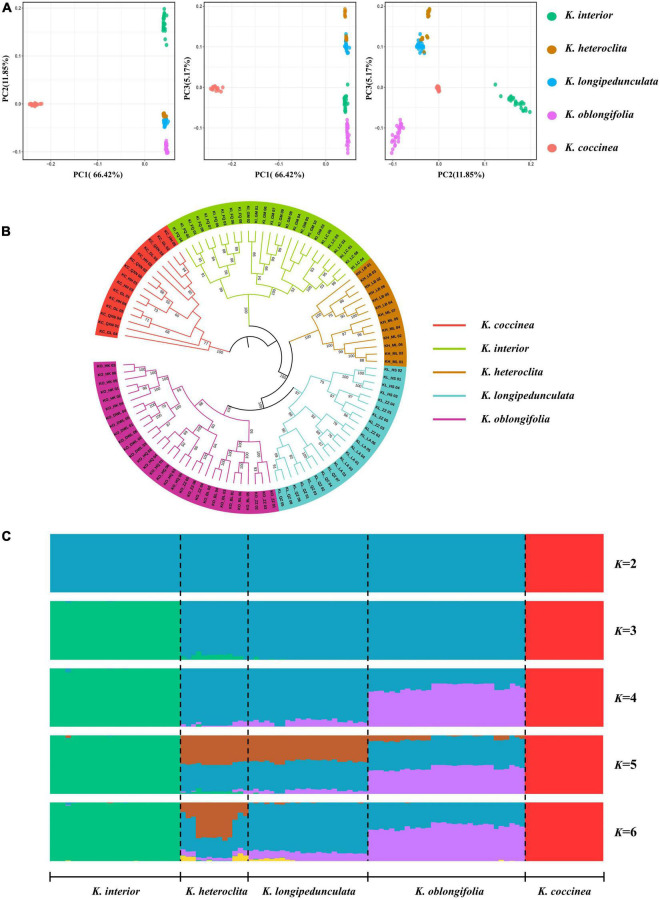
**(A)** PCA plots of the first three components. PC1, PC2, and PC3 are abbreviations for the first three principles. Individuals of different species are represented in different colors. **(B)** The maximum-likelihood tree is based on SNPs. Individuals of different species are represented in different colors. The numbers on the branches are the related bootstrap supports. **(C)** STRUCTURE analyses for *K* = 2–6. Each color represents one genetic cluster. The five species are delimited by a dashed black line.

PCA analysis was performed on five species, PC1 vs. PC2 identified four groups and explained 66.42 and 11.85% of the variation, respectively (Figure 2A). *K. heteroclita* and *K. longipedunculata* clustered closely together. *K. interior*, *K. oblongifolia*, and *K. coccinea* formed three separate groups. The results of PC1 vs. PC3 (5.17% of the variation) and PC2 vs. PC3 showed that *K. heteroclita* and *K. longipedunculata* were relatively separate. However, *K. heteroclita* still had individuals embedded in *K. longipedunculata*, while the other species formed separate groups. The PCA analysis indicates that the genetic relationship between *K. heteroclita*, *K. longipedunculata*, and *K. oblongifolia* was relatively close. To further determine the phylogenetic relationships among these species, we constructed maximum-likelihood trees based on SNPs and chloroplast genomes, respectively. *K. coccinea* was chosen as the outgroup. The phylogenetic tree constructed based on SNPs revealed that each species formed a strongly supported monophyletic clade. *K. longipedunculata* and *K. oblongifolia* were sister groups, *K. heteroclita* was the sister group of these two species, and *K. interior* was the basal clade (Figure 2B). The relationship between these species was like PCA analysis. The results based on the chloroplast genomes showed that *K. longipedunculata* and *K. heteroclita* were not monophyletic (Supplementary Figure 1). To understand the population genetic structure of five species, we used 5,998 non-linked SNPs for STRUCTURE analysis. Results from the delta-*K* analysis of the STRUCTURE HARVESTER output indicated that there were most likely five genetic clusters (Supplementary Figure 2), and the results from *K* = 2 to *K* = 6 were showed (Figure 2C). At *K* = 2, *K. coccinea* first diverged from all species and displayed an independent cluster. This indicates that it has a relatively independent genetic background. At *K* = 3, *K. interior* diverged secondly and had a little genetic admixture. At *K* = 4, *K. heteroclita*, *K. longipedunculata*, and *K. oblongifolia* mainly shared two genetic clusters that were distinct from those of the other species. At *K* = 5, the three species added another shared genetic cluster, and the admixture of the individuals of *K. heteroclita* and *K. longipedunculata* was much closer. In summary, for the relationships between these species, all three analyses shown above demonstrate similar patterns.

### Divergence Scenarios and Time Estimation

**FIGURE 3:**
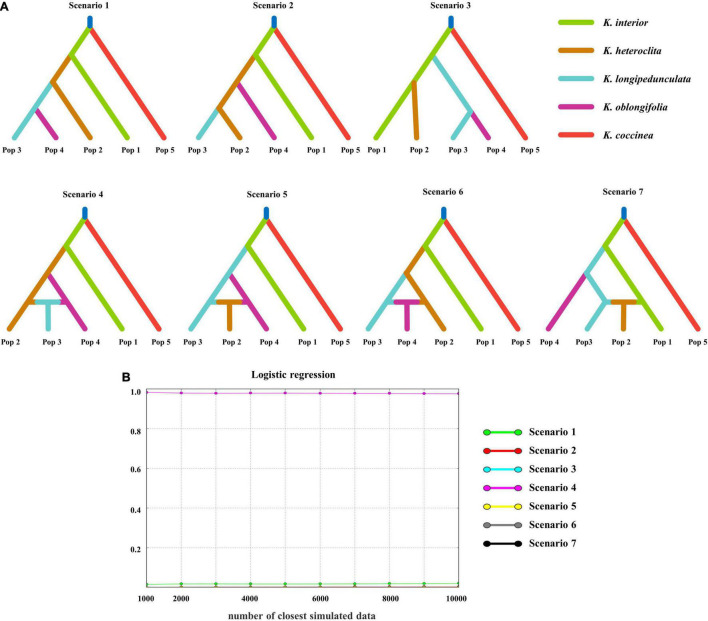
Scenario setting and results of the DIY-ABC analysis. **(A)** Seven divergence scenarios for DIY-ABC analysis. The first three scenarios are without admixture and the latter four are with admixture. **(B)** The posterior probability of different scenarios, scenario 4 has the highest posterior probability.

In the DIY-ABC analysis, scenarios 1–3 were set without admixture events and scenarios 4–7 were set with admixture events (Figure 3A). The difference between the scenarios is mainly in the relationship between *K. heteroclita*, *K. longipedunculata*, and *K. oblongifolia*. Scenario 4 had the highest posterior probability (0.9993, 95% credible interval: 0.9966–1.0000) (Figure 3B). This scenario showed that *K. coccinea* was separated first, followed by *K. interior*, and *K. longipedunculata* originated from hybrid populations derived from *K. heteroclita* and *K. oblongifolia* (Figure 3A).

The tested topologies and inferred partitioned D-statistics were summarized in Table 4 H1, H2, and H3 were the three species except outgroup. D-stat was the average test statistic, and a negative value meant that H1 was closer to H3 than H2. A positive value meant that H2 was closer to H3 than H1. The *Z* value indicated the significance of the test. In general, the absolute values of *Z* values greater than 3 were often used as critical values. When *K. interior* was designated as H1 lineages, all D-stat were positive and numerically large, while the *Z* values were significantly greater than three. This suggested that there may be gene flow or introgression between *K. heteroclita*, *K. longipedunculata*, and *K. oblongifolia*. At the same time, when *K. longipedunculata* and *K. oblongifolia* were designated as H2 and H3, the D-stat were larger. When *K. interior* was designated as H3, the values for D-stat were small and Z were not significant (Table 4).

**TABLE 4:** D-statistics for gene flow involving four species.

H1	H2	H3	D-stat	*Z*	nABBA	nBABA
KI	KH	KL	0.430580	155.691484	305,371.005	121,548.084
KI	KH	KO	0.432596	146.692555	293,601.332	116,285.881
KI	KL	KH	0.424695	152.149413	305,371.005	123,311.730
KI	KL	KO	0.507243	177.399766	330,944.700	108,194.346
KI	KO	KH	0.423683	145.735847	293,601.332	118,851.852
KI	KO	KL	0.502633	193.282819	330,944.700	109,541.703
KH	KL	KI	−0.007203	−1.311835	121,548.084	123,311.730
KH	KL	KO	0.117639	83.805731	201,319.365	158,938.824
KH	KO	KI	−0.010913	−3.124616	116,285.881	118,851.852
KH	KO	KL	0.121572	80.803696	201319.365	157,675.692
KL	KO	KI	−0.006188	−1.033510	108,194.346	109,541.703
KO	KL	KH	0.013989	4.511166	158,938.824	157,675.692

*KI represents K. interior, KH represents K. heteroclita, KL represents K. longipedunculata, and KO represents K. oblongifolia. D-stat is the average test statistic.*

*Z scores greater than 3 represent a significant value.*

**FIGURE 4:**
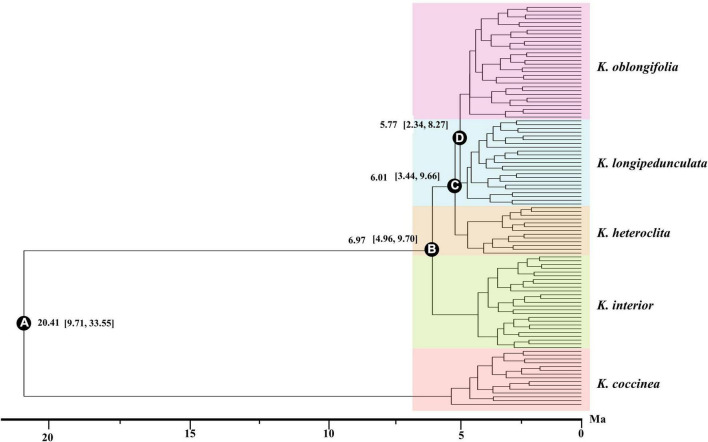
BEAST chronogram of *K. interior* and its related species inferred from the RAD-seq data. The values in brackets represent the 95% highest posterior density intervals of node ages. The letters in the circles correspond to some selected major nodes. The bar below is the time from now, and the unit is Ma.

The divergence time of the five species was estimated with SNAPP, and the results are shown in Figure 4. The topology of the species tree inferred by SNAPP is consistent with the ML tree. The crown age of *Kadsura* was estimated to be 20.41 Ma (95% HPD: 9.71, 33.55 Ma) based on the SNPs dataset. The crown age of the four species was estimated as 6.97 Ma (node B, 95% HPD: 4.96, 9.70 Ma) for *K. interior*, 6.01 Ma (node C, 95% HPD: 3.44, 9.66 Ma) for *K. heteroclita*, 5.77 Ma (node D, 95% HPD: 2.34, 8.27 Ma) for *K. longipedunculata* and *K. oblongifolia*, respectively.

### Ecology Niche Modeling

**FIGURE 5:**
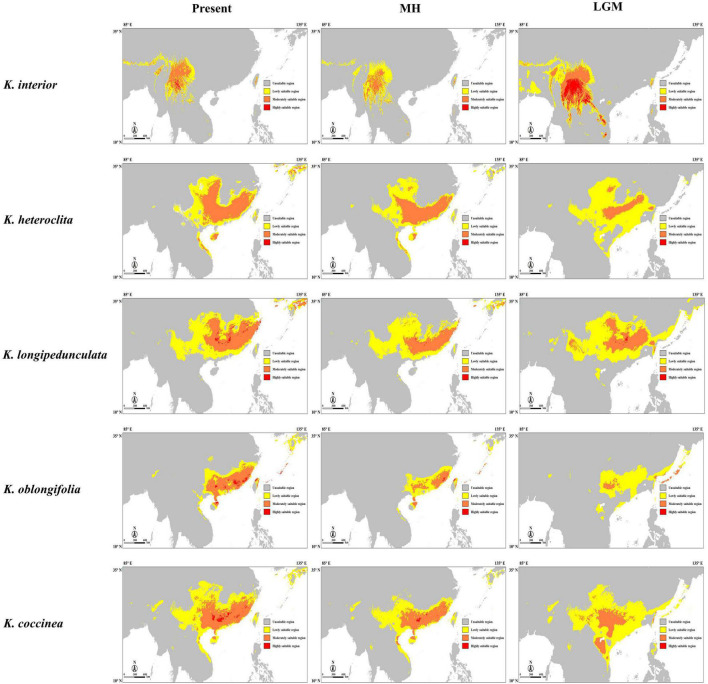
Habitat suitability of *K. interior* and its related species predicted by Maxent for the present, mid-Holocene (MH), and last Maximum Glacial (LGM). The suitable area is divided into three gradients according to the threshold from low to high.

We estimated suitable distribution models for the five *Kadsura* species at three different periods by Maxent, including the last Maximum Glacial (LGM), mid-Holocene (MH), and present (Figure 5). The area values under the AUC of all models were greater than 0.87, indicating that the prediction results were relatively accurate. We used MTSS as the threshold and divided the suitable area into three ranges: low, moderate, and high. From the LGM to present, the total suitable distribution area of *K. interior* showed a change process of contraction, however, the range of moderately and highly suitable areas showed a process of shrinking first and then expanding. Moderately and highly suitable distribution areas of *K. heteroclita* and *K. coccinea* moved to the southeast coast and became more concentrated. From the LGM, the suitable distribution area of *K. oblongifolia* gradually expanded, and there were obvious amplifications in the moderately and highly suitable regions. In general, *K. interior* has the most stable distribution range, with most of the suitable areas in different periods in the southwest of the Yunnan Province and the surrounding areas. The results of the bioclimatic variable contributions analysis showed that the minimum temperature of the coldest month (BIO6) contributed the most to the distribution model of *K. interior*. The annual precipitation (BIO12) contributed the most to the distribution model of *K. oblongifolia*, and precipitation of the warmest quarter (BIO18) contributed the most to the distribution model of the other three species (Supplementary Table 2).

## Discussion

### Phylogenetic Relationship and Genetic Diversity

Several authors have conducted taxonomic revisions on regional or worldwide scales of *Kadsura*, but the treatment of species delimitation under the genus remains controversial (Smith, 1947; Law, 1996; Saunders, 1998; Lin, 2002). For example, Saunders treated *K. interior* and *K. heteroclita* as one species. In Lin’s treatment, *K. interior* was merged into *K. heteroclita* and *K. longipedunculata* was merged into *K. japonica* and the former was adopted by the Flora of China (Xia et al., 2008). In our previous study based on the four gene regions, *K. interior*, *K. heteroclita*, and *K. longipedunculata* were clustered as one clade, and two SNPs could distinguish *K. interior* from the other two species (Guo et al., 2017). This study was based on the SNPs obtained by RAD-seq to explore the relationship between *K. interior* and its related species. The results of PCA showed that, in general, each species clustered into a separate group, but the relationship between *K. heteroclita*, *K. longipedunculata*, and *K. oblongifolia* was relatively close. The populations of *K. interior* and *K. coccinea* remained isolated from the populations of the three species mentioned above. The ML tree showed similar results. The monophyly of each species was strongly supported. *K. interior* was the basal clade of these four closely related species. But the results based on the chloroplast genomes showed that *K. longipedunculata* and *K. heteroclita* were not monophyletic. The incongruent between the RAD-seq and chloroplast sequence in phylogeny may be due to introgression or incomplete lineage sorting.

The population genetic differentiation referred to the obvious difference in the allele frequency between species populations. Generally, both genetic drift and the selection process can cause genetic differentiation between populations. At present, *F*_ST_ is one of the important indicators for detecting genetic differentiation between populations (Wright, 1943). Wright pointed out that *F*_ST_ from 0 to 0.05 indicated that there was no difference between populations, *F*_ST_ from 0.05 to 0.15 suggested that there was moderate differentiation between populations, and *F*_ST_ greater than 0.15 showed that there was high differentiation between populations (Wright, 1978). According to this standard, the *F*_ST_ calculated in this study showed that there was a moderate degree of differentiation among *K. heteroclita*, *K. longipedunculata*, and *K. oblongifolia*, and there was a high degree of genetic differentiation between *K. interior* and the above species. The *F*_ST_ between *K. coccinea* and all other species was extremely large indicating that *K. coccinea* was greatly differentiated from other species. Combining the results of PCA, phylogenetic tree, and various genetic diversity parameters, it can be seen that these five species were relatively independent. We suggest that *K. interior* is a separate species and should not be merged into *K. heteroclita*.

### Population Structure and Divergence Scenarios

The results of the population structure revealed *K* = 5 as the most likely number of clusters best explaining the ancestral components of the five species. When *K* = 5, there were five main genetic clusters in *K. interior* and its related species. The individuals of *K. coccinea* were composed of a consistent genetic cluster. The genetic cluster of the *K. interior* populations was also relatively homogeneous, which was different from the *K. coccinea* populations. This further illustrates the unique genetic background of *K. interior* and the other species. Unlike them, the individuals of *K. heteroclita*, *K. longipedunculata*, and *K. oblongifolia* populations were an admixture of the three genetic clusters, which meant that there may be gene flow or hybridization events among them. Recent studies suggested that the presence of concomitant gene flow during speciation may be a common phenomenon (Nosil, 2008), for example, divergence with gene flow in the incipient speciation of *Miscanthus floridulus* and *M. sinensis* (Huang et al., 2014), and gene flow and divergent selection of two species of *Ipomoea* (Rifkin et al., 2019). These studies indicated that gene flow played an important role in the divergence and eventual speciation of a population. To clarify the specifics of gene flow during the divergence of *K. interior* and its related species, we simulated divergence scenarios and calculated gene flow by DIY-ABC and D-statistics among these species, respectively. The DIY-ABC analysis included three scenarios without admixture and four scenarios with admixture. The best scenario obtained by calculating the posterior probability showed that *K. longipedunculata* split from *K. heteroclita* and *K. oblongifolia*. This meant that there may be gene flow or hybridization events between these three species. The results of the D-stat confirmed this, when *K. interior* was designated as H1 and the other three species were designated as H2 or H3, respectively. The value of D-stat was large and very significant when *K. heteroclita*, *K. longipedunculata*, and *K. oblongifolia* were assigned, respectively, as H1, H2 or H3. The value of D-stat was also positive and the *Z* value was greater than 3. This suggested the existence of gene flow or introgression between *K. heteroclita*, *K. longipedunculata*, and *K. oblongifolia*, but the degree of gene flow between *K. longipedunculata* and *K. oblongifolia* was greater, while the relationship between *K. heteroclita* and *K. longipedunculata* was closer than *K. longipedunculata* and the gene flow was smaller. The phylogenetic tree based on the chloroplast genomes showed that *K. heteroclita* and *K. longipedunculata* were polyphyletic, while the RAD-seq-based phylogenetic tree indicated that the two species were monophyletic. Combined with the analysis of D-statistics, phylogenetic incongruence was more likely to be caused by introgression rather than incomplete lineage sorting.

The morphology of *K. longipedunculata*, *K. oblongifolia*, and *K. heteroclita* has some similarities and transitions, especially in the leaf and flower, and their distribution has overlaps (Law, 1996; Yang, 2006). *K. oblongifolia* is mainly distributed in southern China. From the Hainan Province, Guangdong Province to the Fujian Province, the leaves of *K. oblongifolia* from narrowly lanceolate to narrowly oblong to ovate-lanceolate, showing a transition from narrow to wider. The leaves of *K. longipedunculata* are oblong-lanceolate, obovate-lanceolate, or ovate-oblong. And the shape of the leaves of *K. oblongifolia* in the Guangdong Province and Fujian Province is very similar to that of *K. longipedunculata*. The leaves of *K. heteroclita* are ovate-elliptic to broadly elliptical and wider than *K. longipedunculata*. In the staminate flowers, the receptacle of *K. oblongifolia* is ellipsoid, the top of the receptacle is not elongated, the androecium is spherical, and there are almost no filaments. The receptacle of *K. longipedunculata* is also ellipsoid, but the top is elongated and cylindrical, and does not protrude outside the androecium, and the androecium is spherical. The filaments and the connective are connected into a wide and flat square. The top of the receptacle of *K. heteroclita* is elongated, cylindrical, or conical, protruding outside the androecium, and the androecium is ellipsoid. And the other morphologies are the same as *K. longipedunculata*. The morphological similarity among these species also indicates their close relationship and morphological diversity and transition also indicate the potential gene introgression.

### Divergence Time Estimation and Geohistorical Events Affecting Divergence

The results of the strict molecular clock showed that the divergence of *Kadsura* occurred around the Miocene. The crown age of *K. interior* and its related species was dated to 6.97 Ma. The differentiation time of *K. heteroclita* was dated to 6.01 Ma. The crown ages of *K. longipedunculata* and *K. oblongifolia* were dated to 5.77 Ma. The main uplifting event occurred in the Qinghai–Tibet Plateau (QTP) during 10–8 Ma (Molnar, 2005). At the same time, the Asian interior aridification intensified during 8–6 Ma (An et al., 2001). During this period, aridification was a global phenomenon, marking the climate deterioration from the late Miocene to the Pliocene. The winter monsoon was stronger than before, and the summer monsoon was significantly attenuated (Li and Fang, 1999). The Qinghai–Tibet Plateau growth event and the enhancement of monsoon and inland aridification resulted in major changes in the terrestrial ecosystem in East Asia. During the Miocene period, the southeastern region was significantly affected by the South Asian and East Asian monsoons, and the climate became humid, which may have promoted the diversity of the species in this area (Sun and Wang, 2005). The previous studies illustrated a plateau uplift. The monsoon climate played a key role in the speciation and evolution of the species (Sessa et al., 2012; Xiang et al., 2018; Liu et al., 2019). For example, the west lineage of *Phoebe zhennan* distributed around the Sichuan Basin was affected by the East Asian monsoon and further diversified at ∼7.12 Ma (Xiao et al., 2020). The result of molecular dating indicated that the differentiation time of *K. interior* and its related species was consistent with the main uplifting event of QTP, and the intensifying of Asian interior aridification and monsoon climate. Therefore, the geohistorical events and dramatic climate change should be important factors that lead to the divergence and speciation of *K. interior* and its related species.

### Potential Distribution and Key Bioclimatic Variables

The quaternary glaciation was the most recent glaciation on Earth and was an important stage in the evolution of the Earth’s environment. At that time, most area of Europe and North America was covered by ice sheets, which had devastating effects on biodiversity (Hewitt, 2000). During the quaternary glaciation, only alpine glaciers but no continental glaciers existed in China, which enabled many tertiary plants to survive (Huang et al., 2015). Due to the geological heterogeneity and environmental differences, plants showed different patterns in response to climate change. And the quaternary glaciation and its impact on the changing species distribution and population genetic structure have been topics of intense debate. Some studies suggested that the range of species shrank or fragmented and were preserved in scattered refuges during periods of inhospitable weather. When the climate changed to a favorable period, the range of species expanded or migrated to more suitable latitudes or altitudes (Hewitt, 2004). Habitats are characterized by species with secluded topography and suitable climate. After the glacial period, their distribution tends to shrink as they adapt to the cold climate (Liu et al., 2012). The results of the change in the distribution of *K. interior* in this study were consistent with this pattern. During the LGM, the overall distribution area became larger as the population migrated to lower elevations due to decreasing temperatures. In the MH and present, the population continued to migrate and gather at higher altitudes. The results of the contribution weights of bioclimatic variables to the potential distribution also showed that the minimum temperature in the coldest month was the most important factor affecting the distribution of *K. interior*. Since the LGM, the distribution range of *K. heteroclita*, *K. longipedunculata*, and *K. coccinea* tended to migrate to the southeast, and *K. oblongifolia* experienced significant population expansions. Meanwhile, the results of the contribution weights of bioclimatic variables showed precipitation as the most important factor affecting the distribution of these species, which may also be responsible for such changes in the demographic history of these species. The distribution of *K. longipedunculata* was relatively stable. The results of the ecological niche modeling in showed that *K. interior* is distributed only in Yunnan and its adjacent areas, and *K. oblongifolia* is distributed only in Hainan, Guangdong, and Fujian. *K. heteroclita*, *K. longipedunculata*, and *K. coccinea* are distributed in most parts of southern China. At the same time, from the LGM to the present, the distribution area of *K. oblongifolia* has expanded, and in the process of moving northward, it has spatially overlapped with the populations of *K. longipedunculata* and *K. heteroclita*, which also provide the possibility of introgression among species.

## Conclusion

This study resolved the phylogenetic relationship of *K. interior* and its related species by RAD-seq and suggested that *K. interior* should not be merged into *K. heteroclita*. Furthermore, introgression or hybridization events among the three species *K. heteroclita*, *K. longipedunculata*, and *K. oblongifolia* were found. Previous studies might have overlooked hybridization events among them. In addition, the potential geographic distribution of five species was simulated, which indicated that these species have existed ecological niche differentiation. This study revealed the differences between *K. interior* and its related species from the perspectives of genetics and ecology, which can guide the accurate medication and quality control of similar traditional medicine in *Kadsura*.

## Data Availability Statement

The datasets presented in this study can be found in online repositories. The names of the repository/repositories and accession number(s) can be found below: https://www.ncbi.nlm.nih.gov/, PRJNA792621.

## Author Contributions

HL, XW, and YD conceived and designed the study. YD, XW, JL, and YQ collected the samples. YD, XW, and TQ performed the experiments. YD and XW analyzed the data and drafted the manuscript. PC, HL, and BZ revised the manuscript. All authors contributed to the article and approved the submitted version.

## Conflict of Interest

The authors declare that the research was conducted in the absence of any commercial or financial relationships that could be construed as a potential conflict of interest.

## Publisher’s Note

All claims expressed in this article are solely those of the authors and do not necessarily represent those of their affiliated organizations, or those of the publisher, the editors and the reviewers. Any product that may be evaluated in this article, or claim that may be made by its manufacturer, is not guaranteed or endorsed by the publisher.

## Footnotes

1
https://www.cvh.ac.cn/

2
https://doi.org/10.15468/dl.ubsx7a

3
https://worldclim.org/

## References

[B1] An Z. S. Kutzbach J. E. Prell W. L. Porter S. C. (2001). Evolution of Asian monsoons and phased uplift of the Himalaya-Tibetan plateau since Late Miocene times. *Nature* 411 62–66. 10.1038/35075035 11333976

[B2] Baird N. A. Etter P. D. Atwood T. S. Currey M. C. Shiver A. L. Lewis Z. A. (2008). Rapid SNP discovery and genetic mapping using sequenced RAD markers. *PLoS One* 3:e3376. 10.1371/journal.pone.0003376 18852878 PMC2557064

[B3] Bouckaert R. Vaughan T. G. Barido-Sottani J. Duchêne S. Fourment M. Gavryushkina A. (2019). BEAST 2.5: an advanced software platform for Bayesian evolutionary analysis. *PLoS Comput. Biol.* 15:e1006650. 10.1371/journal.pcbi.1006650 30958812 PMC6472827

[B4] Boukteb A. Sakaguchi S. Ichihashi Y. Kharrat M. Nagano A. J. Shirasu K. (2021). Analysis of genetic diversity and population structure of *Orobanche foetida* populations from Tunisia using RADseq. *Front. Plant Sci.* 12:618245. 10.3389/fpls.2021.618245 33927733 PMC8078179

[B5] Chinese pharmacopoeia commission (2020). *The Pharmacopoeia of the People’sRepublic of China.* Beijing: China Medical And Technology Press.

[B6] Cornuet J. M. Pudlo P. Veyssier J. Dehne-Garcia A. Gautier M. Leblois R. (2014). DIYABC v2.0: a software to make approximate Bayesian computation inferences about population history using single nucleotide polymorphism, DNA sequence and microsatellite data. *Bioinformatics* 30 1187–1189. 10.1093/bioinformatics/btt763 24389659

[B7] Dupin M. Reynaud P. Jarošík V. Baker R. Brunel S. Eyre D. (2011). Effects of the training dataset characteristics on the performance of nine species distribution models: application to *Diabrotica virgifera virgifera*. *PLoS One* 6:e20957. 10.1371/journal.pone.0020957 21701579 PMC3118793

[B8] Durand E. Y. Patterson N. Reich D. Slatkin M. (2011). Testing for ancient admixture between closely related populations. *Mol. Biol. Evol.* 28 2239–2252. 10.1093/molbev/msr048 21325092 PMC3144383

[B9] Earl D. A. Vonholdt B. M. (2012). STRUCTURE HARVESTER: a website and program for visualizing STRUCTURE output and implementing the Evanno method. *Conserv. Genet. Resour.* 4 359–361. 10.1007/s12686-011-9548-7

[B10] Fan J. H. Thien L. B. Luo Y. B. (2011). Pollination systems, biogeography, and divergence times of three allopatric species of Schisandra in North America, China, and Japan. *J. Syst. Evol.* 49 330–338. 10.1111/j.1759-6831.2011.00125.x

[B11] Francis R. M. (2017). pophelper: an R package and web app to analyse and visualize population structure. *Mol. Ecol. Resour.* 17 27–32. 10.1111/1755-0998.12509 26850166

[B12] Fujian Food and Drug Administration (2006). *Chinese Materia Medica Standards of FuJian Province.* Fujian: HaiFeng Press.

[B13] Guangdong Food and Drug Administration. (2004). *Chinese Materia Medica Standards of Guang Dong Province.* Guangzhou: Guangdong Science and Technology Press.

[B14] Guangxi Zhuang Autonomous Region Health Department. (1992). *Chinese Materia Medica Standards of Guang Xi Province.* Nanning: Guangxi Science and Technology Press.

[B15] Guo H. J. Li X. W. Qi Y. D. Wei X. P. Zhang B. G. Liu H. T. (2017). Identification of Dian Ji Xue Teng (*Kadsura interior*) with DNA barcodes. *World J. Tradit. Chin. Med.* 3 11–15. 10.15806/j.issn.2311-8571.2016.0017

[B16] Hewitt G. (2000). The genetic legacy of the Quaternary ice ages. *Nature* 405 907–913. 10.1038/35016000 10879524

[B17] Hewitt G. M. (2004). Genetic consequences of climatic oscillations in the Quaternary. *Philos. Trans. R. Soc. Lond. B Biol. Sci.* 359 183–195. 10.1098/rstb.2003.1388 15101575 PMC1693318

[B18] Hijmans R. J. Cameron S. E. Parra J. L. Jones P. G. Jarvis A. (2005). Very high resolution interpolated climate surfaces for global land areas. *Int. J. Climatol.* 25 1965–1978. 10.1002/joc.1276

[B19] Hill M. P. Hoffmann A. A. Mccoll S. A. Umina P. A. (2012). Distribution of cryptic blue oat mite species in Australia: current and future climate conditions. *Agric. For. Entomol.* 14 127–137. 10.1111/j.1461-9563.2011.00544.x

[B20] Huang C. L. Ho C. W. Chiang Y. C. Shigemoto Y. Hsu T. W. Hwang C. C. (2014). Adaptive divergence with gene flow in incipient speciation of *Miscanthus floridulus/sinensis* complex (Poaceae). *Plant J.* 80 834–847. 10.1111/tpj.12676 25237766

[B21] Huang Y. Jacques F. M. Su T. Ferguson D. K. Tang H. Chen W. (2015). Distribution of Cenozoic plant relicts in China explained by drought in dry season. *Sci. Rep.* 5:14212. 10.1038/srep14212 26369980 PMC4572930

[B22] Jackman S. D. Vandervalk B. P. Mohamadi H. Chu J. Yeo S. Hammond S. A. (2017). ABySS 2.0: resource-efficient assembly of large genomes using a Bloom filter. *Genome Res.* 27 768–777. 10.1101/gr.214346.116 28232478 PMC5411771

[B23] Jakobsson M. Rosenberg N. A. (2007). CLUMPP: a cluster matching and permutation program for dealing with label switching and multimodality in analysis of population structure. *Bioinformatics* 23 1801–1806. 10.1093/bioinformatics/btm233 17485429

[B24] Jiang H. Lei R. Ding S.-W. Zhu S. (2014). Skewer: a fast and accurate adapter trimmer for next-generation sequencing paired-end reads. *BMC Bioinformatics* 15:182. 10.1186/1471-2105-15-182 24925680 PMC4074385

[B25] Kalyaanamoorthy S. Minh B. Q. Wong T. K. F. Von Haeseler A. Jermiin L. S. (2017). ModelFinder: fast model selection for accurate phylogenetic estimates. *Nat. Methods* 14 587–589. 10.1038/nmeth.4285 28481363 PMC5453245

[B26] Kapli P. Yang Z. Telford M. J. (2020). Phylogenetic tree building in the genomic age. *Nat. Rev. Genet.* 21 428–444. 10.1038/s41576-020-0233-0 32424311

[B27] Katoh K. Standley D. M. (2013). MAFFT multiple sequence alignment software version 7: improvements in performance and usability. *Mol. Biol. Evol.* 30 772–780. 10.1093/molbev/mst010 23329690 PMC3603318

[B28] Kearse M. Moir R. Wilson A. Stones-Havas S. Cheung M. Sturrock S. (2012). Geneious Basic: an integrated and extendable desktop software platform for the organization and analysis of sequence data. *Bioinformatics* 28 1647–1649. 10.1093/bioinformatics/bts199 22543367 PMC3371832

[B29] Korunes K. L. Samuk K. (2021). pixy: unbiased estimation of nucleotide diversity and divergence in the presence of missing data. *Mol. Ecol. Resour.* 21 1359–1368. 10.1111/1755-0998.13326 33453139 PMC8044049

[B30] Law Y. W. Xia N. H. Saunders R. M. K. (1996). “*Kadsura* Kaempf. ex Juss,” in *Flora Reipublicae Popularis Sinicae*, Vol. 30 (Beijing: Science Press), 232–234.

[B31] Lewis P. O. (2001). A likelihood approach to estimating phylogeny from discrete morphological character data. *Syst. Biol.* 50 913–925. 10.1080/106351501753462876 12116640

[B32] Li H. Durbin R. (2009). Fast and accurate short read alignment with Burrows-Wheeler transform. *Bioinformatics* 25 1754–1760. 10.1093/bioinformatics/btp324 19451168 PMC2705234

[B33] Li J. Fang X. (1999). Uplift of Qinghai-Tibetan Plateau and environmental change. *Chin. Sci. Bull.* 44, 2217–2224. 10.1007/978-94-010-0965-2_2

[B34] Lin Q. (2002). Taxonomic notes on some species of *Kadsura* (Schisandraceae). *Bull. Bot. Res.* 22 399–411.

[B35] Liu J. Wei X. Zhang X. Qi Y. Zhang B. Liu H. (2018). A comprehensive comparative study for the authentication of the *Kadsura* crude drug. *Front. Pharmacol.* 9:1576. 10.3389/fphar.2018.01576 30740055 PMC6357937

[B36] Liu J. Q. Sun Y. S. Ge X. J. Gao L. M. Qiu Y. X. (2012). Phylogeographic studies of plants in China: advances in the past and directions in the future. *J. Syst. Evol.* 50 267–275. 10.1111/j.1759-6831.2012.00214.x

[B37] Liu M. L. He Y. L. López-Pujol J. Jia Y. Li Z.-H. (2019). Complex population evolutionary history of four cold-tolerant Notopterygium herb species in the Qinghai-Tibetan Plateau and adjacent areas. *Heredity* 123 242–263. 10.1038/s41437-019-0186-2 30742051 PMC6781143

[B38] Mallet J. Besansky N. Hahn M. W. (2016). How reticulated are species? *Bioessays* 38 140–149. 10.1002/bies.201500149 26709836 PMC4813508

[B39] Miller M. R. Dunham J. P. Amores A. Cresko W. A. Johnson E. A. (2007). Rapid and cost-effective polymorphism identification and genotyping using restriction site associated DNA (RAD) markers. *Genome Res.* 17 240–248. 10.1101/gr.5681207 17189378 PMC1781356

[B40] Minh B. Q. Schmidt H. A. Chernomor O. Schrempf D. Woodhams M. D. Von Haeseler A. (2020). IQ-TREE 2: new models and efficient methods for phylogenetic inference in the genomic era. *Mol. Biol. Evol.* 37 1530–1534. 10.1093/molbev/msaa015 32011700 PMC7182206

[B41] Molnar P. (2005). Mio-Pliocene growth of the Tibetan Plateau and evolution of East Asian climate. *Palaeontol. Electron.* 9 1–23. 10.1175/jcli-d-21-0569.1

[B42] Nosil P. (2008). Speciation with gene flow could be common. *Mol. Ecol.* 17 2103–2106. 10.1111/j.1365-294X.2008.03715.x 18410295

[B43] Paetzold C. Wood K. R. Eaton D. A. R. Wagner W. L. Appelhans M. S. (2019). Phylogeny of Hawaiian *Melicope* (Rutaceae): RAD-seq resolves species relationships and reveals ancient introgression. *Front. Plant Sci.* 10:1074. 10.3389/fpls.2019.01074 31608076 PMC6758601

[B44] Pritchard J. K. Stephens M. Donnelly P. (2000). Inference of population structure using multilocus genotype data. *Genetics* 155 945–959. 10.1093/genetics/155.2.945 10835412 PMC1461096

[B45] Ren G. Mateo R. G. Liu J. Suchan T. Alvarez N. Guisan A. (2017). Genetic consequences of Quaternary climatic oscillations in the Himalayas: *Primula tibetica* as a case study based on restriction site-associated DNA sequencing. *New Phytol.* 213 1500–1512. 10.1111/nph.14221 27696413

[B46] Rifkin J. L. Castillo A. S. Liao I. T. Rausher M. D. (2019). Gene flow, divergent selection and resistance to introgression in two species of morning glories (*Ipomoea*). *Mol. Ecol.* 28 1709–1729. 10.1111/mec.14945 30451335

[B47] Rochette N. C. Catchen J. M. (2017). Deriving genotypes from RAD-seq short-read data using Stacks. *Nat. Protoc.* 12 2640–2659. 10.1038/nprot.2017.123 29189774

[B48] Rochette N. C. Rivera-Colón A. G. Catchen J. M. (2019). Stacks 2: analytical methods for paired-end sequencing improve RADseq-based population genomics. *Mol. Ecol.* 28 4737–4754. 10.1111/mec.15253 31550391

[B49] Saunders R. M. K. (1998). Monograph of *Kadsura* (Schisandraceae). *Syst. Bot. Monogr.* 54 24–106. 10.2307/25096646

[B50] Sessa E. B. Zimmer E. A. Givnish T. J. (2012). Phylogeny, divergence times, and historical biogeography of New World *Dryopteris* (Dryopteridaceae). *Am. J. Bot.* 99 730–750. 10.3732/ajb.1100294 22434775

[B51] Smith A. C. (1947). The families Illiciaceae and Schisandraceae. *Sargentia* 7 1–224. 10.5962/p.265318 33311142

[B52] Soraggi S. Wiuf C. Albrechtsen A. (2018). Powerful Inference with the D-statistic on low-coverage whole-genome data. *G3* 8 551–566. 10.1534/g3.117.300192 29196497 PMC5919751

[B53] Sritalahareuthai V. Temviriyanukul P. On-Nom N. Charoenkiatkul S. Suttisansanee U. (2020). Phenolic Profiles, Antioxidant, and Inhibitory Activities of *Kadsura heteroclita* (Roxb.) Craib and *Kadsura coccinea* (Lem.) A.C. Sm. *Foods* 9:1222. 10.3390/foods9091222 32887386 PMC7555767

[B54] Stange M. Sánchez-Villagra M. R. Salzburger W. Matschiner M. (2018). Bayesian divergence-time estimation with genome-wide single-nucleotide polymorphism data of sea catfishes (ariidae) supports miocene closure of the panamanian isthmus. *Syst. Biol.* 67 681–699. 10.1093/sysbio/syy006 29385552 PMC6005153

[B55] Sun X. Wang P. (2005). How old is the Asian monsoon system?—Palaeobotanical records from China. *Palaeogeogr. Palaeoclimatol. Palaeoecol.* 222 181–222. 10.1016/j.palaeo.2005.03.005

[B56] Wright S. (1943). Isolation by distance. *Genetics* 28 114–138. 17247074 10.1093/genetics/28.2.114 PMC1209196

[B57] Wright S. (1978). *Evolution and the Genetics of Populations.* Chicago: University of Chicago Press.

[B58] Wu K. M. Farrelly J. G. Upton R. Chen J. (2007). Complexities of the herbal nomenclature system in traditional Chinese medicine (TCM): lessons learned from the misuse of *Aristolochia*-related species and the importance of the pharmaceutical name during botanical drug product development. *Phytomedicine* 14 273–279. 10.1016/j.phymed.2006.05.009 16863692

[B59] Xia N. H. Liu Y. H. Richard M. K. S. (2008). *Flora of China: SCHISANDRACEAE.* Beijing: Science Press.

[B60] Xiang K. L. Erst A. S. Xiang X. G. Jabbour F. Wang W. (2018). Biogeography of *Coptis Salisb*. (Ranunculales, Ranunculaceae, Coptidoideae), an Eastern Asian and North American genus. *BMC Evol. Biol.* 18:74. 10.1186/s12862-018-1195-0 29793422 PMC5968522

[B61] Xiao J. H. Ding X. Li L. Ma H. Ci X. Q. Van Der Merwe M. (2020). Miocene diversification of a golden-thread nanmu tree species (*Phoebe zhennan*, Lauraceae) around the Sichuan Basin shaped by the East Asian monsoon. *Ecol. Evol.* 10 10543–10557. 10.1002/ece3.6710 33072279 PMC7548194

[B62] Xu J. Liu J. S. Li B. Wei X. P. Qi Y. D. Zhang B. G. (2022). Comparison of blood tonic efficacy and chemical constituents of *Kadsura interior* A.C. Smith and its closely related species. *Chin. Med.* 17:14. 10.1186/s13020-021-00544-w 35039063 PMC8762946

[B63] Xu J. Wei X. P. Liu J. S. Qi Y. D. Zhang B. G. Liu H. T. (2021). Genome sizes of four important medicinal species in *Kadsura* by flow cytometry. *Chin. Herb. Med.* 13 416–420. 10.1016/j.chmed.2021.05.002 PMC9476794 36118928

[B64] Yang Z. Rannala B. (2012). Molecular phylogenetics: principles and practice. *Nat. Rev. Genet.* 13 303–314. 10.1038/nrg3186 22456349

[B65] Yang Z. R. (2006). *Systematics of Schisandraceae.* Doctor’s thesis. Beijing: The Institute of Botany, Chinese Academy of Sciences.

[B66] Yin M. Z. Peng H. S. Cheng M. E. (2017). Textual research on Fengqing *Jixueteng* (*Kadsura interior* A. C. Smith), Kunming *Jixueteng* (*Millettia dielsiana* Harms ex Diels) and *Jixueteng* (*Spatholobus suberectus* Dunn). *Zhonghua Yi Shi Za Zhi* 47 342–347. 10.3760/cma.j.issn.0255-7053.2017.06.004 29374946

[B67] Zhang J. Chen M. Dong X. Lin R. Fan J. Chen Z. (2015). Evaluation of four commonly used DNA barcoding Loci for chinese medicinal plants of the family schisandraceae. *PLoS One* 10:e0125574. 10.1371/journal.pone.0125574 25938480 PMC4418597

[B68] Zhou M. Yang G. Sun G. Guo Z. Gong X. Pan Y. (2020). Resolving complicated relationships of the *Panax bipinnatifidus* complex in southwestern China by RAD-seq data. *Mol. Phylogenet. Evol.* 149:106851. 10.1016/j.ympev.2020.106851 32438045

