# Supplementary material for: RAD-Seq and Ecological Niche Reveal Genetic Diversity, Phylogeny, and Geographic Distribution of Kadsura interior and Its Closely Related Species
Source: Front Plant Sci. 2022 Apr 26;13:857016. doi: 10.3389/fpls.2022.857016 (PMC9087809; doi:10.3389/fpls.2022.857016)
Supplement: Supplementary file 1 [file Data_Sheet_1.docx]

Supplementary Material

**Supplementary Table 1.** Fixation index *Fst* at the population level

|  | KI_GM | KI_LC | KH_LB | KH_ML | KL_HS | KL_ZZ | KL_QZ | KL_LA | KO_ZZ | KO_BL | KO_HQ | KO_DWL | KO_HK | KC_HH | KC_QXN | KC_GL |
| --- | --- | --- | --- | --- | --- | --- | --- | --- | --- | --- | --- | --- | --- | --- | --- | --- |
| KI_FQ | 0.0795 | 0.1121 | 0.2101 | 0.1707 | 0.2090 | 0.2171 | 0.1940 | 0.1884 | 0.1962 | 0.1909 | 0.2234 | 0.2363 | 0.2142 | 0.6695 | 0.6639 | 0.6549 |
| KI_GM |  | 0.0857 | 0.1901 | 0.1631 | 0.1947 | 0.2011 | 0.1841 | 0.1736 | 0.1865 | 0.1820 | 0.2084 | 0.2187 | 0.2031 | 0.6526 | 0.6471 | 0.6388 |
| KI_LC |  |  | 0.2365 | 0.1904 | 0.2387 | 0.2444 | 0.2146 | 0.2108 | 0.2174 | 0.2127 | 0.2430 | 0.2602 | 0.2359 | 0.6843 | 0.6813 | 0.6724 |
| KH_LB |  |  |  | 0.1522 | 0.1524 | 0.1631 | 0.1425 | 0.1194 | 0.1536 | 0.1500 | 0.1753 | 0.1828 | 0.1716 | 0.6670 | 0.6589 | 0.6499 |
| KH_ML |  |  |  |  | 0.1531 | 0.1562 | 0.1527 | 0.1348 | 0.1478 | 0.1403 | 0.1742 | 0.1878 | 0.1716 | 0.6379 | 0.6320 | 0.6257 |
| KL_HS |  |  |  |  |  | 0.1447 | 0.1302 | 0.1219 | 0.1367 | 0.1309 | 0.1694 | 0.1805 | 0.1657 | 0.6710 | 0.6627 | 0.6572 |
| KL_ZZ |  |  |  |  |  |  | 0.1170 | 0.1211 | 0.1368 | 0.1408 | 0.1635 | 0.1741 | 0.1650 | 0.6745 | 0.6670 | 0.6575 |
| KL_QZ |  |  |  |  |  |  |  | 0.1114 | 0.1131 | 0.1407 | 0.1184 | 0.1182 | 0.1147 | 0.6212 | 0.6175 | 0.6072 |
| KL_LA |  |  |  |  |  |  |  |  | 0.1268 | 0.1239 | 0.1529 | 0.1634 | 0.1474 | 0.6449 | 0.6391 | 0.6324 |
| KO_ZZ |  |  |  |  |  |  |  |  |  | 0.1057 | 0.1063 | 0.1142 | 0.1026 | 0.6253 | 0.6229 | 0.6117 |
| KO_BL |  |  |  |  |  |  |  |  |  |  | 0.1342 | 0.1426 | 0.1227 | 0.6364 | 0.6291 | 0.6201 |
| KO_HQ |  |  |  |  |  |  |  |  |  |  |  | 0.1103 | 0.1024 | 0.6333 | 0.6273 | 0.6163 |
| KO_DWL |  |  |  |  |  |  |  |  |  |  |  |  | 0.1105 | 0.6462 | 0.6404 | 0.6311 |
| KO_HK |  |  |  |  |  |  |  |  |  |  |  |  |  | 0.6258 | 0.6214 | 0.6131 |
| KC_HH |  |  |  |  |  |  |  |  |  |  |  |  |  |  | 0.1007 | 0.1099 |
| KC_QXN |  |  |  |  |  |  |  |  |  |  |  |  |  |  |  | 0.1084 |

**Supplementary Table 2.** The contribution weights of bioclimatic variables to the potential distribution areas

| **Species** | **Variable** | **Description** | **Percent contribution** |
| --- | --- | --- | --- |
| *K. interior* | BIO6 | Min temperature of the coldest month | 34.40% |
| *K. heteroclita* | BIO18 | Precipitation of warmest quarter | 69.10% |
| *K. longipedunculata* | BIO18 | Precipitation of warmest quarter | 71.90% |
| *K. oblongifolia* | BIO12 | Annual precipitation | 67% |
| *K. coccinea* | BIO18 | Precipitation of warmest quarter | 68.50% |


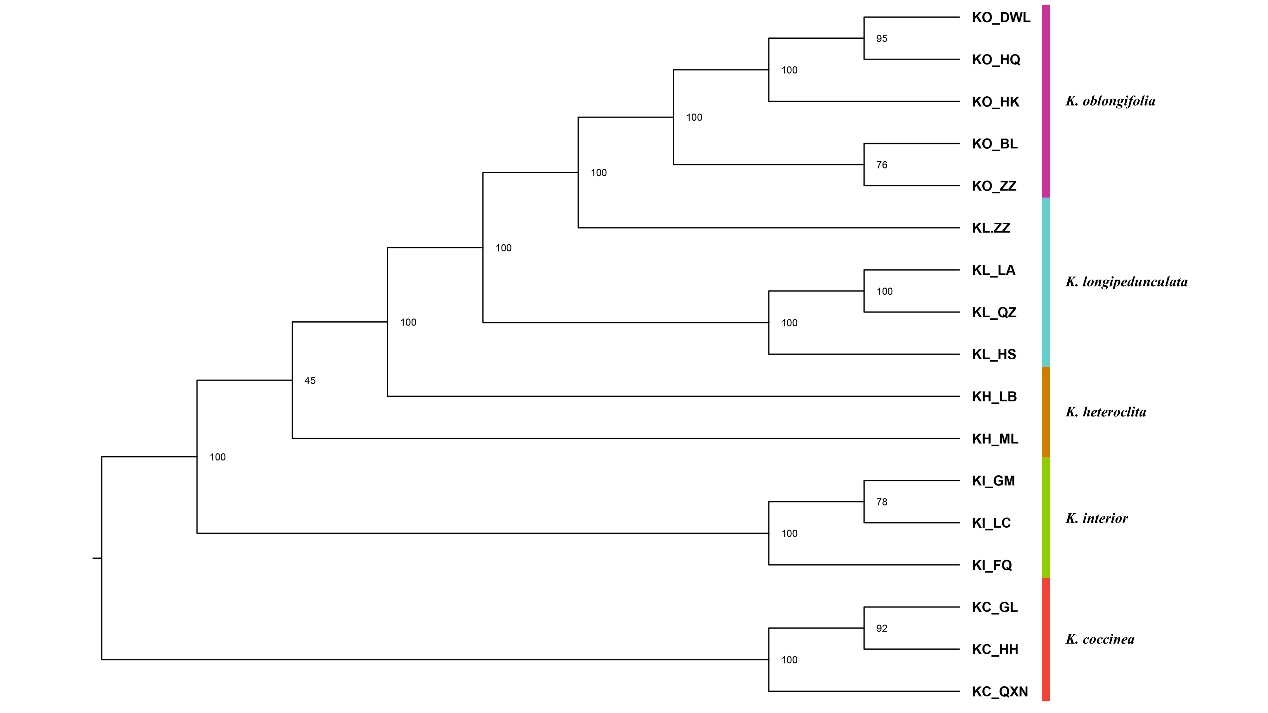


**Supplementary Figure 1.** Maximum-likelihood tree based on chloroplast genome of 17 populations. Numbers on branches are the related bootstrap supports.


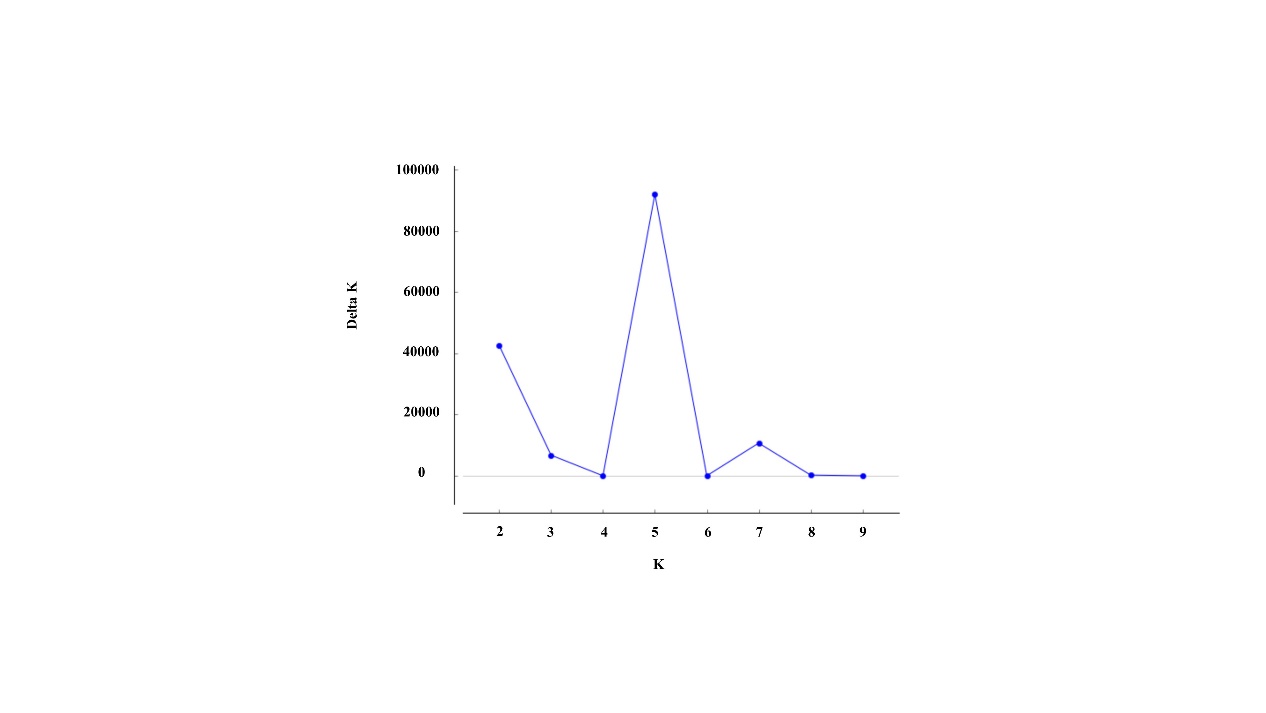


**Supplementary Figure 2.** Delta-*K* at *K*=2-9. Delta-*K* maximizes when K=5
